# Lung-Based, Exosome Inhibition Mediates Systemic Impacts Following Particulate Matter Exposure

**DOI:** 10.3390/toxics10080457

**Published:** 2022-08-07

**Authors:** Keegan Lopez, Alexandra Camacho, Quiteria Jacquez, Mary Kay Amistadi, Sebastian Medina, Katherine Zychowski

**Affiliations:** 1 Department of Biology, College of Arts and Sciences, New Mexico Highlands University, Las Vegas, NM 88901, USA; 2 College of Nursing, University of New Mexico-Health Sciences Center, Albuquerque, NM 87131, USA; 3 Arizona Laboratory for Emerging Contaminants, University of Arizona, Tucson, AZ 85721, USA

**Keywords:** metals, particulate matter, lung, brain, inflammation

## Abstract

Particulate matter (PM) exposure is a global health issue that impacts both urban and rural communities. Residential communities in the Southwestern United States have expressed concerns regarding the health impacts of fugitive PM from rural, legacy mine-sites. In addition, the recent literature suggests that exosomes may play a role in driving toxicological phenotypes following inhaled exposures. In this study, we assessed exosome-driven mechanisms and systemic health impacts following inhaled dust exposure, using a rodent model. Using an exosome inhibitor, GW4869 (10 μM), we inhibited exosome generation in the lungs of mice via oropharyngeal aspiration. We then exposed mice to previously characterized inhaled particulate matter (PM) from a legacy mine-site and subsequently assessed downstream behavioral, cellular, and molecular biomarkers in lung, serum, and brain tissue. Results indicated that CCL-2 was significantly upregulated in the lung tissue and downregulated in the brain (*p* < 0.05) following PM exposure. Additional experiments revealed cerebrovascular barrier integrity deficits and increased glial fibrillary acidic protein (GFAP) staining in the mine-PM exposure group, mechanistically dependent on exosome inhibition. An increased stress and anxiety response, based on the open-field test, was noted in the mine-PM exposure group, and subsequently mitigated with GW4869 intervention. Exosome lipidomics revealed 240 and eight significantly altered positive-ion lipids and negative-ion lipids, respectively, across the three treatment groups. Generally, phosphatidylethanolamine (PE) and phosphatidylcholine (PC) lipids were significantly downregulated in the PM group, compared to FA. In conclusion, these data suggest that systemic, toxic impacts of inhaled PM may be mechanistically dependent on lung-derived, circulating exosomes, thereby driving a systemic, proinflammatory phenotype.

## 1. Introduction

As a result of U-mining cessation following the Cold War era, hundreds of former U-mines were left abandoned in the Southwestern United States. Proximity to abandoned U-mine and mill sites is a significant risk factor that contributes to negative health effects in surrounding communities [1,2,3,4]. These mines were often left unremediated, resulting in contamination of the surrounding soil, water, and air with toxic metals, including U, V, As, and Ni [5]. Inhalation of fugitive, wind-blown dusts from these mine-sites is currently under investigation in relation to neurovascular and neurological disease [5,6].

The previous literature has demonstrated that mine-based dust is mechanistically more toxic than background (control) dust and may potentiate cardiopulmonary toxicity [7,8]. The biological mechanisms driving this impact may be due to circulating factors that interact with the endothelium [6]. White blood cells may extravasate through the activated endothelium via diapedesis and eventually form foam cells between the endothelial wall and the smooth muscle layer [9], thereby driving atherogenic disease. In the case of neuroinflammatory disease, the blood–brain barrier (BBB) is the primary target for these secondary circulating factors, subsequent to inhaled toxicants [10]. Activation of glial and other cells, such as astrocytes and microglia, may occur after inhaled toxicant exposure. Neurological disorders have recently been described in association with PM exposures. Silica-based dusts may not only drive pulmonary inflammation, but also cause neuroinflammatory impacts in an autoimmune model [5]. Furthermore, mental health impacts and depression have been linked to short-term exposure to PM and a depressive-like response in mice due to inflammation in the brain [11,12]. More specifically, there is a neuro- and immunologic association among stress, inflammation, and air pollution exposures [13,14,15,16]. Furthermore, circulating factors, such as exosomes, have been used in experimental clinical models and may have prognostic value for brain and mental health disorders [17]. However, specific molecular mechanisms driving this effect are currently unknown.

The “secretome” is an ongoing area of research, with regard to toxicological exposures [6,10,18,19]. These secondary factors may modify the circulation by altering adhesion molecules on the endothelium surface [20]. Extracellular vesicles (EVs) are lipid-based vesicles secreted by cells into the extracellular and interstitial space [21,22]. Extracellular vesicles contain a lipid bilayer membrane that protects the internal material including, but not limited to peptides, miRNAs, metabolites, and nucleic acids. Furthermore, extracellular vesicles are involved in intercellular communication and cellular disposal, and they may act as circulating biomarkers. There are several different subclasses of EVs, including microvesicles (MVs), apoptotic bodies, and exosomes, which are distinguished on the basis of biogenesis, composition, and size. Exosomes, also known as intraluminal vesicles (ILVs), are some of the smallest EVs and typically span 30–150 nm in diameter. Exosomal cargo varies and may also depend on exosomal biogenesis-type [23,24,25]. Exosome and extracellular vesicle-mediated exposures have been linked to a number of pathologies including neurodegeneration, pulmonary disease, infertility, and cancer [25,26,27,28]. Endosomal sorting complexes required for transport (ESCRT)-dependent pathways mediate ubiquinated cytosolic proteins as cargo; however, ubiquinated proteins and peptides are not required for ESCRT-independent selection, which includes a wide array of proteins including MHC receptors, MMPs, β-catenin, and viral-associated protein [29]. The ESCRT-independent pathway involves ceramides and neutral sphingomylinase, both involved in microvesicle budding.

Lipids are critical components of extracellular vesicles; however, our knowledge of the composition and function of these molecules is limited, despite the fact that circulating lipids are key drivers of neurovascular disease [30]. Plasma membrane disruption is crucial to allow exosome formation. This modification of the outer membrane promotes EV formation. Multiple lipid pathways are involved in exosome biogenesis including ESCRT, ceramide, hexadecylglycerol, PIP_3_, cholesterol, and phosphatidic acid pathways [31]. Exosome composition, including a high ratio of protein/lipids, may enhance membrane rigidity, ensuring durability to circulate within biological fluids [32].

The objective of this study was to examine the mechanistic role of lung-derived exosomes following PM exposure and impacts on the neurovascular system using a rodent model. We hypothesized that PM induces proinflammatory lung-derived exosomes which eventually circulate systemically and cause inflammatory impacts in the brain such as reduced barrier integrity in cerebrovascular endothelial cells and activation of astrocytes.

## 2. Materials and Methods

### 2.1. Animal Care and Study Design

**Figure 1:**
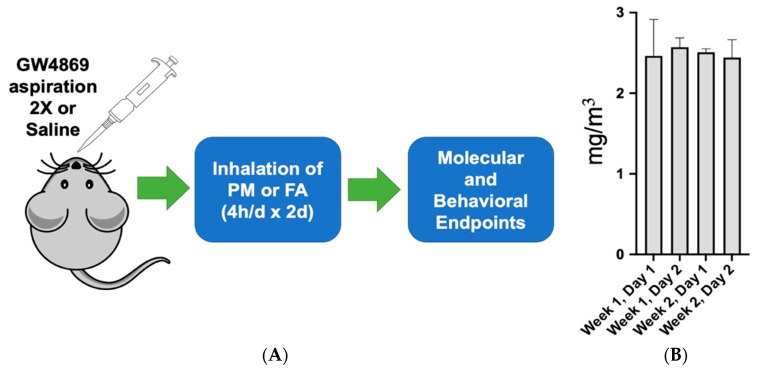
Study design and PM concentration. (**A**) Mice were subjected to 50 μL of GW4869 (10 μM) or 50 μL of saline via oropharyngeal aspiration the day before and the day of exposure to either FA or PM (2.51 ± 0.08 mg/m^3^). (**B**) PM concentration each exposure day (2 days total).

Mice were ordered from Taconic Biosciences (Albany, NY, USA) and acclimated in appropriate housing facilities for 2 weeks, as per the university-approved institutional animal care and use committee (IACUC) protocol. C57BL/6 mice were randomly assigned to three different treatment groups (*n* = 10 mice per group): HEPA-filtered air (Lab Products LLC, Seaford, DE) + saline (FA), particulate matter + saline (PM), or PM + an exosome inhibitor, GW4869 (MilliporeSigma, St. Louis, MO, USA) (PM + GW4869) (Figure 1A). Firstly, 50 μL of 10 μM GW4869 or 1× physiological saline solution (PSS, MilliporeSigma, St. Louis, MO) was instilled into the lungs of mice 1 h prior to inhaled exposure of mine-dust from St. Anthony mine PM exposure or FA prior to each 4 h exposure.

### 2.2. Inhalation Exposures

Previously characterized dust samples, derived from St. Anthony mine [5], a former commercial mine near Paguate, NM, were sieved to <60 μm to facilitate aerosolization in a closed inhalation chamber. A whole-body, bench-scale exposure system with air supply, dilution, and filtration with real-time TSI monitoring for particulate concentration and size distribution was used to for all rodent exposures. Gravimetric filter samples were collected to confirm true mass calculations. The average chamber concentration was measured over several days and averaged 2.51 ± 0.08 mg/m^3^ (Figure 1B).

### 2.3. Bronchoalveolar Lavage Fluid Following Exposure

A small cannula was inserted using an incision in the trachea of each mouse, while under isoflurane anesthesia. Bronchoalveolar lavage fluid (BALF) was extracted by flushing the lungs with 1 mL of phosphate-buffered saline (PBS) and withdrawn using a sterile syringe for collection. Total cell, neutrophil, and macrophage counts were then subsequently assessed, using Hoechst staining (ThermoFisher Scientific, Waltham, MA, USA) techniques [33] for dead/live cell acquisition using a hemocytometer.

### 2.4. BALF Exosome Isolation and Quantification

Exosomes isolated from bronchoalveolar lavage fluid (BALF) were centrifuged at 3000× *g* for 15 min to remove cellular debris, and then subsequently transferred into a new tube. The appropriate amount of ExoQuick solution was added incubated with the BALF using a commercially available kit, ExoQuick ULTRA EV Isolation Kit (System Biosciences, Palo Alto, CA, USA). The solution and the BALF were mixed well and incubated overnight. The ExoQuick/BALF mixture was centrifuged at 1500× *g* for 30 min. Centrifugation was performed at room temperature or 4 °C. The supernatant was carefully aspirated following centrifugation at 1500× *g* for 5 min. The remaining, precipitated EV pellet was resuspended in 350 µL of Lysis Buffer, vortexed for 15 s, and allowed to sit at room temperature for 5 min. The quantification and the confirmation of exosomes in the sample were conducted using Nanocyte technology according to previously published literature [34].

### 2.5. ICP-MS Metals Content in BALF Exosomes

Isolated exosomes were digested using a hot-block technique digestion following methods outlined in Meyer et al. (2018) [35] using a Digi Prep hot block and PP digestion tubes (SCP Science, Quebec, CA, USA). Mass-based aliquots were treated with aliquots of concentrated nitric acid and hydrogen peroxide (J.T. Baker ULTREX II Ultrapure). Samples were heated to 90 °C to denature proteins, resulting in a clear solution. The digestion was then diluted to achieve 2% acid prior to analysis by ICP-MS. All samples were analyzed on an Agilent Model 7700× ICP-MS (Santa Clara, CA, USA). The instrument parameters were as follows: RF power = 1450 W, dwell time = 50 ms, sweeps per replicate = 100, nebulizer flow = 0.95 L/min, coolant = 15 L/min, auxiliary = 1.3 L/min, sample uptake = ~0.400 mL/min. Replicates were run in technical triplicate according to each sample, and the acquisition mode was set to peak hopping.

The US EPA protocol in Method 6020 was used. Each run included quality control checks referred to as Initial Calibration Verification (ICV) standards and Independent Calibration Verification. The QC checks fell within ±10% of their expected value. After calibration, after every 10 samples, and at the run end, quality control samples were reanalyzed. These QC checks included a mid-range standard (Continuing Calibration Verification, CCV), a QC solution sample from an independent source, and the matched matrixed sample. Examples are NIST 1643 trace metals in water or a certified reference material (High Purity Standards, Charleston, SC, USA). Results were within 25% of the expected value.

### 2.6. RT-qPCR Gene Expression

Gene expression was assessed using real-time quantitative polymerase chain reaction (RT-qPCR) methods. Lung and brain tissues were dissected and snap-frozen in liquid nitrogen. Samples were then subsequently stored in a −80 °C freezer for further use. Total RNA was extracted using a commercial RNA kit (RNeasy, Qiagen, Germantown, MD, USA). Samples were later thawed and reverse-transcribed using High-Capacity cDNA Reverse Transcription reagents (Applied Biosystems, Foster City, CA, USA), prior to performing qPCR. Gene expression was assessed using Taqman Gene Expression protocols and a 384 CFX Opus Real-Time PCR System (Bio-Rad, Hercules, CA, USA). Genes assessed included CCL-2 (Mm00441242_m1), TGF-β (Mm0178820_m1), IL-6 (Mm00446190_m1), TNFα (Mm00443258_m1), CXCL-1 (Mm04207460_m1), and IL-1β (Mm00434228_m1); TATA-binding protein (TBP, Mm01277042) was used as a housekeeping control gene (Thermo Fisher Scientific, Waltham, MA, USA). Relative gene expression using these target genes was analyzed using the 2^−∆∆CT^ method [36].

### 2.7. Electric Cell Impedance Sensing Assay

Transendothelial resistance of mouse cerebrovascular endothelial cells (mCECs) in response to both serum and isolated exosomes was examined using the electric cell impedance sensing (ECIS) system (Applied Biophysics, Troy, NY, USA). This system utilizes an electrical current to examine alterations in endothelial cell integrity, according to Ohm’s law (V = IR). In this study, 5% serum was incubated with a monolayer of mCECs that were plated and grown to confluence, as monitored by a plateau in transendothelial resistance. Transendothelial resistance was measured at 16 k Hz, and the normalized adhesion index was calculated on the basis of previous literature [37]. In a second set of experiments, exosomes were isolated from the FA and PM exposure groups, and cell regrowth was monitored at 16 kHz after an electrical shock (wounding). Normalized resistance was reported as a plateau was reached within the cell culture.

### 2.8. Immunofluorescent Staining

Brains were dissected from each mouse and subsequently halved. The right hemisphere was embedded using optimal cutting temperature (OCT) media. After freezing, the block was sectioned using a cryostat at 60 μm and mounted on positively charged slides. Fluorescent staining was conducted using commercially available reagents (Abcam, Waltham, MA, USA). Glial fibrillary acidic protein (GFAP) rabbit polyclonal antibody (Abcam, Cambridge, MA, USA) was diluted to 1:2000 and incubated on each slide for 1–2 h. Sections were counterstained with DAPI (Thermo Scientific, Waltham, MA, USA) in a sealed box protected from light for 8 min. Once dry, ProLong Gold antifade reagent (Invitrogen, Waltham, MA, USA) was applied to the tissue. Once complete, slides were stored at room temperature.

### 2.9. Microscopy and Imaging

Images were captured used Leica TSC SP8 Confocal Microscope. DAPI was captured at an excitation peak at 359 nm and an emission peak at 457 nm, while GFAP’s fluorescence was captured at 490 nm excitation and 525 nm emission. Slides were imaged using a 20× oil objective using 4× zoom resulting at 80× magnification. Both hippocampi and the cortex were imaged for each slide. Individual nuclei and astrocytes were imaged along with colocalized regions.

### 2.10. High-Throughput Astrocyte Quantification via HALO Analysis

Image analysis produced from the confocal microscope was performed using the HALO software (Indicalabs, Albuquerque, NM, USA), running on a Dell Precision Tower 7810 PC incorporating dual 3 GHz Intel Xenon processors with 32 GB of RAM. Images were annotated to separate the hippocampi and the cortex by creating separate annotation layers, the hippocampus being annotation layer one and the cortex being annotation layer two. Post segmentation, the image was further annotated to exclude any sections of high density that were not able to be read by the HALO software. The dentate gyrus was excluded from the analysis of every hippocampus, due to its high density of nuclei. After the annotations were set, analysis was run on all of the images. Post-analysis data was analyzed using Graph Pad (GraphPad Holdings, LLC, San Diego, CA, USA). Parameters measured from visualized astrocytes included the GFAO area of both hippocampi and the cortex (μm^2^), percentage area, inner and outer area, minimum diameter, maximum diameter, optical density (OD), colocalized area (μm^2^), and percentage of GFAP stained cells (object 2) colocalized with DAPI (object 1).

### 2.11. Behavioral Tasks

Behavioral tasks including assays related to learning and spatial memory, stress and anxiety, or learning and spatial memory were conducted following inhaled exposures. Four tasks were assessed including the open-field test and O-maze, both measures of stress and anxiety, and the novel object test and Y-maze, two tests of learning and spatial memory, sequentially across a span of 4 days. All behavioral studies were executed at the University of New Mexico, Health Sciences Center, Center for Brain Recovery and Repair Preclinical Core, by the same user. The total duration of interactions and the time spent in each designated area were tracked using Noldus Ethovision XT software to quantify both learning and memory deficits and overall stress and anxiety, over the course of a 5 min behavioral task.

### 2.12. Serum Exosome Isolation

Serum was separated from blood and stored at −80° until further use. Serum was centrifuged at 2000 rpm for 30 min to remove any excess debris. The supernatant containing clarified serum was then transferred to a new tube using the ultracentrifugation method [38]. Serum and reagent were mixed by pipetting and incubated at ~4 °C for 30 min. After incubation, the sample was centrifuged at 10,000 rpm for 10 min at room temperature. The supernatant was then aspirated. The remaining exosome pellet was then resuspended with 200 μL of 1× PBS. The sample was then transferred to an Exosome Purification Column and centrifuged at 3000 rpm for 10 min at 4 °C. The filtrates were collected, transferred to a new tube, and stored at −80° until further use.

### 2.13. Sample Preparation for Lipidomics

Serum exosome sample preparation was executed by adding 1.2 mL of chloroform/MeOH (2:1, *v/v*) and 0.2 mL of ultrapure water into the sample and vortexed, before centrifuging for 10 min at 3000 rpm at 4 °C. The dried exosomes were resuspended in 200 μL of isopropyl alcohol/MeOH (1:1, *v/v*) with 5 μL of LPC (12:0) as an internal standard. The sample was then centrifuged for 10 min at 12,000 rpm at 4 °C, and the supernatant was used for LC–MS analysis.

### 2.14. UPLC–MS and Data Analysis for Lipidomics

Separation was performed by UPLC (Thermo, Ultimate 3000 LC). The mobile phase consisted of solvent A (60% ACN + 40% H_2_O + 10 mM HCOONH_4_) and solvent B (10% ACN + 90% isopropyl alcohol + 10 mM HCOONH_4_) with a gradient elution (0–10.5 min, 30–100% B; 10.5–12.5 min, 100% B; 12.5–12.51 min, 100–30% B; 12.51–16 min, 30% B). The mobile phase flow rate was 0.3 mL/min. The column temperature was maintained at 40 °C. ESI+ and ESI− modes were set at the following parameters: ESI+: heater temperature, 300 °C; sheath gas flow rate, 45 arb; auxiliary gas flow rate, 15 arb; sweep gas flow rate, 1 arb; spray voltage, 3.0 kV; capillary temperature, 350 °C; S-lens RF level, 30%. ESI−: heater temperature, 300 °C; sheath gas flow rate, 45 arb; auxiliary gas flow rate, 15 arb; sweep gas flow rate, 1 arb; spray voltage, 3.2 kV; capillary temperature, 350 °C; S-lens RF level, 60%. During the sample analysis, three quality control (QC) samples were run in triplicate to avoid changes in chromatographic retention time and signal intensity. Lipidomics data were analyzed and heatmaps were generated using python packages. Both positive- and negative-ion serum-borne exosomal lipidomics were reported.

### 2.15. Statistical Analyses

A one-way ANOVA was executed for the majority of experimental data, with Tukey’s post hoc test. A *p*-value <0.05 was considered statistically significant in all cases. Significant values are accordingly indicated with an asterisk (*) in the figures. Data in the figures are represented as the mean ± standard error of the mean (SEM).

## 3. Results

### 3.1. Bronchoalveolar Lavage Cells

**Figure 2:**
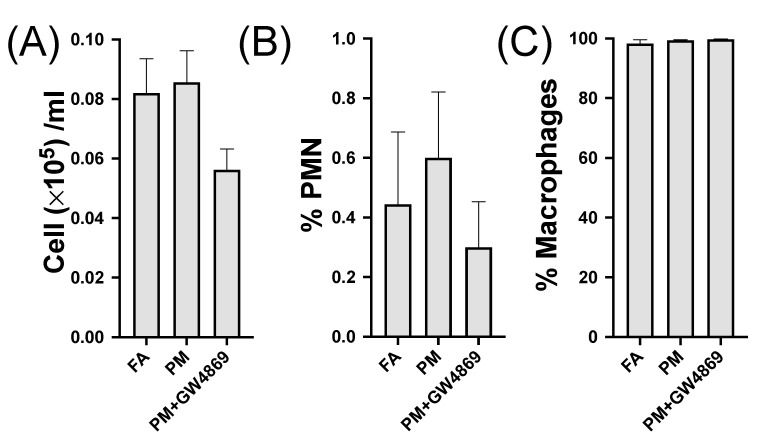
Bronchoalveolar lavage fluid. Cells from lung fluid from FA, PM, and PM + GW4869 inhibitor including (**A**) total cells, (**B**) polymorphonuclear neutrophils (% PMN), and (**C**) macrophages (% macrophages). A one-way ANOVA was performed with a Kruskal–Wallis post hoc test, and *p* ≤ 0.05 was considered significant. Data represent the mean ± SEM.

Lavage results indicated no significant changes in total cells, %PMN, and %macrophages (Figure 2). No statistically significant changes were noted among the FA, PM, and PM + GW4869 groups. However, these modest cellular changes were likely caused by the delay due to the series of behavioral tests that occurred between PM exposure and euthanasia.

### 3.2. Brain and Lung Gene Expression

**Figure 3:**
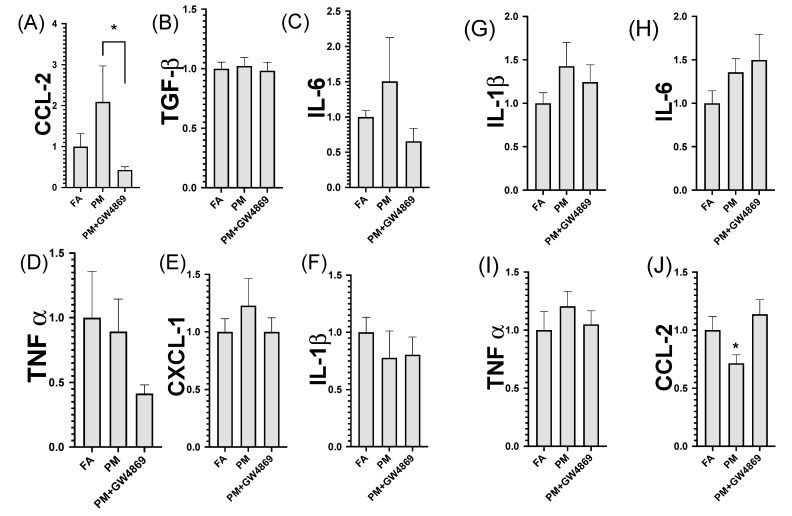
Lung and brain RT-qPCR whole-lung homogenate gene expression for RT-qPCR analysis (**A**–**F**). Brain homogenate gene expression (**G**–**J**). Data were statistically analyzed using a one-way analysis of variance test (ANOVA), followed by a Kruskal Wallis post hoc test; *p* ≤ 0.05 was considered statistically significant, as indicated by the asterisk (*). Data represented include the mean ± SEM.

Lung CCL-2 was significantly diminished in the PM exposure group, relative to FA and PM + GW4869 (*p* = 0.01, Figure 3). Lung gene expression indicated no significant changes in TGF-β, IL-6, TNFα, CXCL-1, and IL-β, according to mRNA RT-qPCR analysis. Genes tested in the right brain whole hemisphere indicated no statistically significant changes in IL-β, TNFα, and IL-6, according to a one-way ANOVA (≤0.05). Interestingly, CCL-2 was significantly diminished in the brain (*p* = 0.03) following PM exposure and this impact was mitigated via administration of GW4869.

### 3.3. GFAP Staining and Cerebrovascular Endothelial Integrity

**Figure 4:**
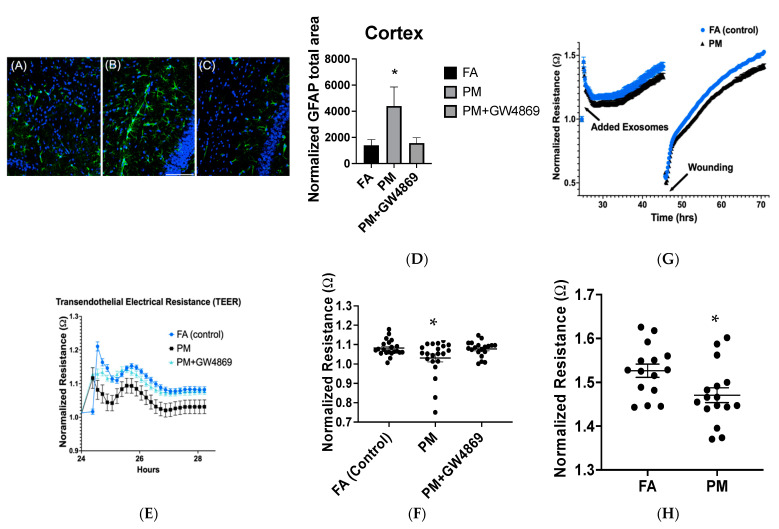
Cerebrovascular integrity. Representative images of brain cortex sections (**A**–**C**) stained with DAPI (nuclear staining, blue) and normalized GFAP staining (green) in FA, PM, and PM + GW4869 treatment groups with respective quantification (**D**). Transendothelial electrical cell impedance sensing (ECIS) in cerebrovascular endothelial cells treated with 5% serum from the aforementioned exposure treatment groups (**E**,**F**). There was significant (*p* = 0.02) diminished resistance in vascular endothelial integrity following serum treatment, suggesting endothelial leakage in the PM group, which was mitigated with GW4869, according to a one-way ANOVA. (**G**,**H**) Transendothelial resistance following treatment with solely *exosomes* and cell culture wounding (scratch assay) with regrowth (*p* = 0.01). Data are considered significant at *p* ≤ 0.05, as indicated by the asterisk (*). Data represent the mean ± SEM.

Hippocampus GFAP staining did not indicate any changes in GFAP staining according to a number of assessed parameters including optical density (OD) and total normalized GFAP area. Normalized GFAP total area was significantly upregulated (*p* = 0.03) in the PM exposure group, relative to FA and PM + GW4869 treatment (Figure 4). Other parameters tested including astrocyte percentage area, inner and outer area, minimum, maximum, and median diameter, and optical density (OD), which in this study, did not significantly change among treatment groups. This impact was significantly mitigated with the administration of GW4869 in the PM exposure group and mimicked GFAP staining similar to the FA control group.

Endothelial cerebrovascular integrity indicated significantly diminished transendothelial electrical resistance (TEER) in the PM exposure, relative to FA and PM + GW4869 exposure groups, following 5% serum incubation with cerebrovascular endothelial cells, according to normalized resistance measurements. In the secondary experiment assessing serum-derived exosomes on cerebrovascular wound healing, normalized resistance diminished in the PM exposure group, relative to FA.

### 3.4. Behavioral Tasks

**Figure 5:**
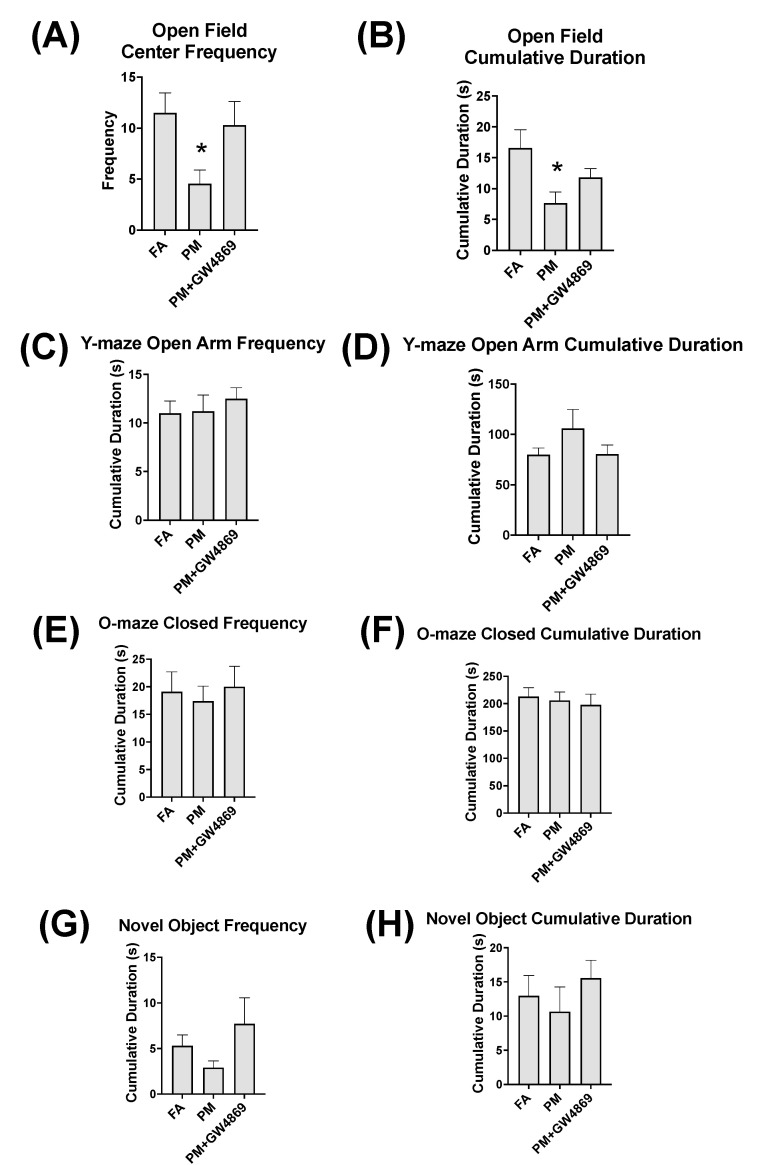
Behavioral tasks. Behavioral tasks were executed following FA, PM, and PM + GW4869 aspiration. These included the open-field test (**A**,**B**), Y-maze (**C**,**D**), O-maze (**E**,**F**), and novel object test (**G**,**H**). The open-field test resulted in significantly diminished center frequency visitation (*p* = 0.0482) and open-field cumulative duration (*p* = 0.0407). Data represent the mean ± SEM. Data are considered significant at *p* ≤ 0.05, as indicated by the asterisk (*).

Behavioral tasks including the open-field test resulted in a significant change in the PM exposure group, relative to the FA and PM + GW4869 treatment groups (Figure 5). The open-field test resulted in significantly diminished center frequency visitation (*p* = 0.0482) and open-field cumulative duration (*p* = 0.0407) following PM exposure. The Y-maze, O-maze, and novel object frequency and cumulative duration remained unchanged among FA, PM, and PM + GW4869 and did not result in any significant differences.

### 3.5. Exosome-Lipidomics and Exosome Composition

**Figure 6:**
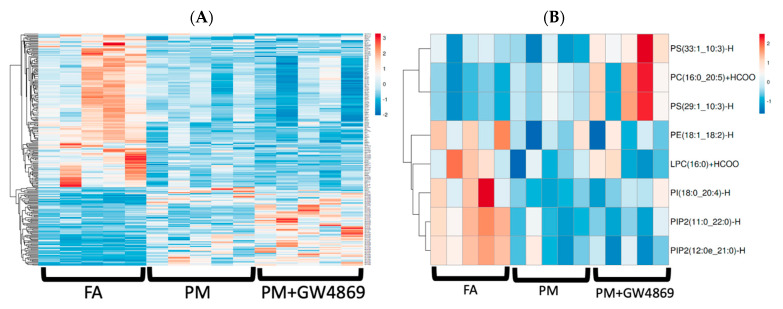
Exosome lipidomics. (**A**) Positive-ion, serum-borne exosome lipidomics. (**B**) Negative-ion serum-borne exosome lipidomics. For lipids with a VIP value >1.5, differential expression demonstrated in the heatmaps was based on a one-way ANOVA analyzed between treatment groups. Statistically significant lipids were determined at *p* ≤ 0.05.

The majority of positive-ion downregulated lipids in the PM, compared to FA, consisted of PC and PE lipids, while a significant number of upregulated lipids in the PM group were triglycerides (TG) (Figure 6). Lipid composition did not change significantly with GW4869 aspiration, and exosome lipid composition remained relatively consistent between PM and PM + GW4869. In a second set of experiments, well-documented toxic metals including As, V, U, Pb, Sb, and Sn that were assessed in isolated BALF exosomes presented either negligible or no detectable level in the FA and PM samples examined (Supplementary Figure S1).

## 4. Discussion

There is an established link among particulate matter, associated metals, neurotoxicity, and mental health outcomes [39]. Furthermore, the role of exosomes and other extracellular vesicles following air pollution is an ongoing area of study [22,27]. In this study, we examined the mechanistic role of lung-based exosomes in a preclinical, rodent model exposed to an environmentally relevant dust sample, derived from the St. Anthony mine-site in New Mexico.

Bronchoalveolar lavage fluid results did not indicate significant changes among treatment groups, which was likely due to the delay between exposure and euthanasia. Other PM studies indicated an increase in BAL cells immediately following PM exposure, which resolved over time. Metal-based PM exposure induces biological changes (lipids) via exosomes; however, toxic metals were not detected in exosomes following PM exposure (Supplementary Figure S1). Determining exosome composition using novel analytical methods, such as lipidomics, is an emerging field in research, and further exploration of exosome composition is warranted in order to understand pathology and contribution to disease development. Our results suggest that pulmonary inflammation largely resolves several days (5 days) following acute dust exposure at the concentrations we used (2 days, 2.51 ± 0.08 mg/m^3^). CCL-2, however, was still upregulated in the lung even 5 days after exposure (upon euthanasia). This lung CCL-2 upregulation was mitigated with GW4869 administration, which may suggest a mechanistic, proinflammatory role of lung-driven exosomes following PM exposure. Interestingly, we found CCL-2 mRNA expression diminished in the brain (cortex) in the PM exposure group alone. This may have been a compensatory response to PM exposure, as several behavioral tasks were performed between exposure and euthanasia, resulting in a slight delay in organ collection. Activated cerebrovascular endothelial cells and astrocytes may release chemokines such as CCL-2 and CXCL-1, which in turn stimulate microglia in the brain [40]. This contributes to the proinflammatory cascade and subsequent neurological impairment [41]. These results indicate that cerebrovascular integrity and subsequent astrocyte activation may be mediated by lung-derived exosomes following inhaled PM, as depicted in Figure 4. Other studies have noted that extracellular vesicles may mediate cerebrovascular integrity [42], and these inflammatory vesicles may modulate pericyte cellular status [43].

There is an emerging role for lipid-based research in air pollution studies [44]. Recent studies suggest that O_3_-mediated specialized pro-resolving lipid mediators (SPMs) may drive O_3_-induced pulmonary inflammation. The majority of the literature has focused on differential miRNA expression in exosomes [23,26]; however, using untargeted lipidomics, we demonstrated significant downregulation in predominantly PE and PC exosome lipids following inhaled PM exposure. These two lipid classes are the most abundant fatty acids and are present in all mammalian cell membranes. Abnormally high or low PC or PE values have been linked with disease progression, with regard to energy metabolism, including changes in mitochondria energy production [45]. Downregulation of PE and PC lipids has been implicated in neurodegenerative diseases such as Alzheimer’s disease and other related dementias [46]. Lipids in Alzheimer’s disease are under current investigation as biomarkers, with the ultimate intention of finding therapeutic targets [47].

Our data also suggest the role of exosomes in driving cerebrovascular impairment following PM exposure (Figure 4), which is mitigated with GW4869 lung-based administration. These data further define the role of exosomes and the lung–brain axis following PM exposures. Our exosome lung-based blockade indicates that cerebrovascular cells may be susceptible to exosomes post PM exposure. Interestingly, this lung-based exosome blockade also suggests that circulating exosomes following PM exposure may have an impact on transcriptional (mRNA) changes in CCL-2. Our results suggest that CCL-2 upregulation following PM exposure in the lung was mitigated with GW4869 oropharyngeal aspiration (Figure 3). In addition, CCL-2 was significantly downregulated in the brain following PM exposure; however, this may have been a compensatory response, due to the delay between PM exposure and euthanasia, during behavioral studies. Prior air pollution studies have examined the role of the BBB in mediating secondary molecular impacts following exposures [6,10]. Other factors, such as the serum peptidome have been examined extensively throughout air pollution studies [48]. Upregulation of aortic CCL-2 has also been implicated following combination exposures such as fine particulate matter and gases [49]. However, this study is the first to suggest that exosome inhibition via the ESCRT-independent pathway may impact CCL-2 transcription following PM exposures. Future studies examining differential roles of exosome biogenesis following PM exposure are warranted.

## 5. Conclusions

Under these conditions, we mechanistically determined that exosomes, derived from the lung, may play a role in neuroinflammatory impacts following inhaled PM exposures. Furthermore, CCL-2 may play a role in exosome-driven impacts following PM exposures. We also determined that the lipid composition of exosomes is significantly altered by PM exposure mainly via PE and PC lipids. Further research is warranted to determine mechanisms of exosome alterations following air pollution exposures and subsequent systemic consequences.

## Data Availability

Data are available upon reasonable request.

## Supplementary Materials

The following are available online at https://www.mdpi.com/article/10.3390/toxics10080457/s1, Figure S1: BALF exosome characterization. Metal levels (μg/g) in isolated BALF exosomes (Left) Nanocyte characterization of BALF exosomes after saline (control) or GW4869 oropharyngeal aspiration (Right), demonstrating significant exosome inhibition following GW4869 aspiration.
