# Supplementary material for: Lung-Based, Exosome Inhibition Mediates Systemic Impacts Following Particulate Matter Exposure
Source: Toxics. 2022 Aug 7;10(8):457. doi: 10.3390/toxics10080457 (PMC9413489; doi:10.3390/toxics10080457)
Supplement: Supplementary file 1 [file toxics-10-00457-s001.zip › toxics-1817783-supplementary.pdf]

toxics

Supplementary Materials: Lung-Based, Exosome Inhibition Mediates Systemic Impacts Following Particulate Matter Exposure

Keegan Lopez, Alexandra Camacho, Quiteria Jacquez, Mary Kay Amistadi, Sebastian Medina and Katherine Zychowski

	As	V	U	Pb	Sb	Sn
FA	0	0	0	0.01	0	0
FA	0	0	0	0.01	0	0
FA	0	0	0	0.01	0	0
PM	0	0	0	0.01	0	0
PM	0	0	0	0.02	0	0
PM	0	0	0	0.01	0	0

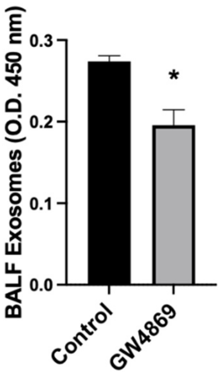

Figure S1. BALF exosome characterization. Metal levels (µg/g) in isolated BALF exosomes (Left) Nanocyte characterization of BALF exosomes after saline (control) or GW4869 oropharyngeal aspiration (Right), demonstrating significant exosome inhibition following GW4869 aspiration. Data are considered statistically significant at $p \leq 0.05$ and indicated by an asterisk (*).
